# Bibliographical Notices

**Published:** 1838-01-01

**Authors:** 


### Periscope;

OR,

### CIRCUMSPECTIVE REVIEW.

"Ore trahit quodcunque potest, atque addit acervo."

### Notices of some New Works.

An Essay on Pyrexia, or Symptomatic Fever, as illustrative of the Nature of Fever in General. By *Henry Clutterbuck*, M.D. 8vo. Highley, 1837.

Our readers are not to be informed at this time of day, that ever since the first publications of Dr. Clutterbuck and M. Broussais, the medical world has been a good deal divided respecting the nature and seat of fever. One party looks upon every fever as having some local focus of irritation or inflammation, cognizable or incognizable, whence the pyrexial symptoms radiate to every part of the machine by sympathy. The other party still maintains that the phenomena of fever may be produced by some general impression on the system, as for example, by malaria, without any necessary dependence on topical inflammation. If Dr. Clutterbuck and M. Broussais had agreed on the "local habitation" of fever, the coincidence would have been a strong corroboration of their doctrine, but unfortunately the Briton fixed on the brain, while the Frenchman determined on the gastro-intestinal canal as the seat of the irritation or phlogosis. When, indeed, we reflect on the numerous moral causes which disturb the sensorial functions in man, and the wide class of both moral and physical causes which derange the digestive functions in the same animal, we need not wonder that the followers of Clutterbuck and Broussais should find ample proofs, during life and after death, of the ravages of fever, both in the brain and intestinal canal. Upon far fewer facts than they possess, many an ingenious theory has been built, to yield, in its turn, to some more powerful successor. But, after all, when we observe the phenomena of intermittent fever—the type of continued fever—we can hardly reconcile them with the existence of topical inflammation *as the cause of the phenomena.*

Dr. Clutterbuck having, in a former work, traced the cause of what is called continued or idiopathic fever, to inflammation of the brain, has now completed his task by treating of the various symptomatic fevers, or those which are obviously dependent on inflammation of one or more parts of the body. This procedure cuts away all ground for controversy, because all have long been agreed that the symptomatic fevers are the consequences of topical phlogosis. There is, however, one point left open for litigation. Holding to the doctrine that all fevers depend on local inflammation, the Doctor maintains that when we cannot detect the seat of the pyrexia, as in the chest, abdomen, or head, we are, nevertheless to infer that it exists, and act accordingly. This is a somewhat heavy tax on our faith.

"The object of the following pages is to endeavour to shew that a *febrile* state of system, or what is technically denominated *symptomatic fever*, is always a

*secondary* affection, the result of inflammation, and of no other cause. In this respect, the subject is of importance for the purpose of *diagnosis*, as it may lead us to discover the existence of inflammation in cases where it might not otherwise be suspected, that is, in cases where the local signs are not obvious, or with difficulty to be observed. It is not unimportant, likewise, in regard to practice; for although *pyrexia* be but a secondary state, it is capable of influencing materially both the primary disease (the inflammation which produced it) and the general system. Its influence on the primary disease is sometimes favourable, sometimes the reverse, according to circumstances that will be pointed out. It is often the cause of greater suffering to the patient than the local disease that gave rise to it; and is sometimes the more immediate cause of the fatal termination, as in the case of *pulmonary consumption*. Persons dying of this most fatal of diseases, are more frequently destroyed by the the *hectic fever* (one of the forms of *pyrexia*), and the disorder this occasions in functions that are essential to the continuance of life, than by the absolute destruction of the lungs themselves; which are seldom so much or so extensively injured in their structure, as to be altogether incapable of carrying on their peculiar function in a manner adequate to the support of life. Hence, our efforts are most usefully directed towards controlling the *hectic* symptoms, for over the local disease we have little power." *Introd.* vii.

Symptomatic pyrexia, characterized by *heat of skin, frequency of pulse,* and *foulness of tongue,* has never, Dr. C. maintains, been accurately distinguished by systematic writers from what are called idiopathic fevers. We must admit, indeed, that there is no one symptom in general fever, which may not occasionally present itself in symptomatic fever. The prostration of strength is usually greater in idiopathic than in symptomatic pyrexia, and this fact is brought forward by our author as a strong support of his doctrine that the brain (the source of voluntary power) is the seat of the former disease. We are ready to confess that there is something in this argument.

General fever has always been represented as consisting of three stages, more or less marked—the cold, the hot, and the sweating stage—all being concluded, in general, within the twenty-four hours. Dr. C. maintains that the same phenomena obtain in the symptomatic fevers. Certainly in hectic fever there is very often found a daily paroxysm of the three stages alluded to, and in most inflammations, say pneumonia, there is a cold stage or shivering, at the beginning, succeeded by re-action, and occasionally terminating, or rather remitting in slight moisture on the skin. But Dr. Clutterbuck himself acknowledges that the hot stage lasts some hours—days—or even *weeks*—the pyrexia being modified by exacerbations and remissions. He thinks, however, that the three stages commonly bear some proportion to each other, even in the symptomatic fevers. No doubt when the rigor is very severe and long-continued, we may expect a tremendous re-action, and probably perspiration :—but when the chilly stage is scarcely perceptible, we have afterwards a long fever, little varied, for some weeks. In such cases, the proportion seems very much like disproportion.

We quote the following passage as evincing accurate observation at the bedside of sickness.

"The change in the appearance of the tongue is one of the most remarkable of these, and the most deserving of attention; because it serves, better than any other single sign, to mark the slightest degrees of *febrile action.* For in numerous instances, where neither the *pulse* is quickened nor the *heat of skin* sensibly increased, the existence of *pyrexia,* or a *febrile state,* is shewn by some or other of the changes in the appearance of the tongue now to be mentioned.

Whenever a *febrile* state of system arises, whatever be the cause, the appearance of the tongue undergoes a remarkable change. In most cases, its upper surface (and this only), instead of the usual florid colour which belongs to it in health, becomes covered, to a greater or less extent, with an apparent crust, or

*fur*, as it is called*, that differs in thickness, colour, and other respects, at different times, so as to afford the most important indications, not merely of the existence of *pyrexia* (and therefore of its cause, *inflammation*), but likewise, in many cases, of the degree and quality of the inflammation, the danger that attends it, and, sometimes, even of the particular seat it occupies in the body.

In slight *febrile* disorders, that are apparently without danger, the fur on the tongue is of small extent, and confined to the middle and back parts of its surface; and it is also generally thin, and light in colour. If the disease be on the increase, the fur on the tongue becomes thicker and more extensive, so as often to cover the whole of its upper surface. It also acquires in such cases a darker hue; and if the disease is likely to prove fatal, the colour changes to a dark yellow, or brown, and, in certain cases, to absolute blackness. Generally speaking, the drier the tongue is, the more active and violent is the existing inflammation.

On some occasions, as in certain varieties of *idiopathic* fever, there is no observable fur on the tongue; its appearance, nevertheless, is very different from that of health, so as to mark sufficiently the *febrile state*. It becomes in such cases preternaturally red, as if deprived of its cuticle, and resembles raw flesh; and it is attended with soreness, so as to become painful, when irritating matter of any kind is applied to it.

When the *liver* is affected by inflammation, and the disease is so situated as to interrupt, or even to impede considerably, the passage of the bile into the intestine, the crust on the tongue is of a yellowish or *bilious* appearance, thus indicating the seat of the inflammation. When, again, the cerebral substance is generally or extensively inflamed, as in *simple* fever of the *typhoid kind*, the tongue turns more or less brown, according to the degree and danger of the disease. Nothing, in fact, is more characteristic of *proper* or *idiopathic* fever, than this brown appearance of the tongue; and when, as frequently happens, *proper fever* (brain affection) arises in the course of *pulmonic* inflammation, or any other, the complication is marked by the change in the appearance of the tongue here mentioned; accompanied by a manifestly disordered state of the *sensorial* functions, in addition to the proper pulmonary symptoms.

As a furred tongue is an unequivocal sign of existing *pyrexia*, or *febrile action* (as this again is of inflammation), so its disappearance is a criterion of returning health that is rarely found to disappoint us. And when important organs are suffering from inflammation, a patient can hardly be considered safe, so long as much foulness of the tongue remains; nor ought we, in such circumstances, to relax in our endeavours to give relief.

The crust on the tongue sometimes disappears gradually, and almost imperceptibly; at other times, the separation is effected abruptly; the crust cracks and peels off in flakes; presenting a ragged appearance, and often leaving the surface beneath excoriated and sore.

I may remark here, that the indications afforded by this state of the tongue have often been mistaken, and the appearance referred to a wrong source. It has been, and, indeed, still is, a prevailing opinion (though only of late years, since the *stomach* has been looked to as the great focus of disease), that the tongue is, as it were, an *index* to the stomach; and that its appearance is but a counterpart, or type, of that which exists in the latter organ. It seems to be imagined, that where the tongue is foul and coated, a similar condition exists in the stomach, if not throughout the alimentary canal. It has been even thought that, in the case of *aphthæ* or *thrush*, the same white vesicular appearance that is observed in the mouth and fauces, extends, at times, throughout the whole ali-

* "It is not, however, a deposition of foul matter on the tongue, but an alteration of its natural covering, the cuticle."

mentary canal; and this is considered to be proved by a similar appearance being observed at the extremity of the rectum. Of this, however, there is no proof. The peculiar appearance observed in the mouth and fauces, in cases of *aphthæ*, is an affection of the skin, and of the cuticle covering it: but these structures can hardly be traced beyond the fauces. Inflammation no doubt often exists, in such cases, throughout the mucous membrane of the intestines; as indicated by pain, and soreness, and an abundant and acrid secretion from the inflamed surfaces: but this is very different from the fur on the tongue that accompanies the *febrile state* simply, and which commonly takes place without any corresponding disorder of the stomach or intestinal canal. The tongue, in short, is an *index* to the *sanguiferous system*, the excited and disordered state of which it clearly serves to mark; and not to the *stomach*, as commonly supposed. This is a point of some importance, practically considered. The employment of *purgatives*, and other means directed particularly to the stomach, on the latter supposition, is, doubtless, often highly useful, as tending to relieve inflammation when it really exists, and, by so doing, clears the tongue; but the objection is, that such treatment is not always adequate to the purpose, and requires the aid of more active remedies, and of *blood-letting* more especially, which cannot always be safely dispensed with." 17.

The various secretions are much changed and disordered in pyrexia—generally diminished. Hence the dryness of the mouth and skin, the constipation of the bowels, the paucity of urine, and the dark colour of the motions.

In the second section, Dr. C. adverts to the changes observed in the blood during pyrexia, but offers nothing that is not well known. In the third Section he adverts to the varieties of pyrexia. They may exist in all degrees—from states in which the pulse, tongue, secretions, &c. are scarcely altered, in any perceptible degree, from their natural condition, up to the most violent synocha. These varieties depend, of course, on age, temperament, the nature of the cause, and the structure of the part inflamed.

Section four takes up the *causes* of pyrexia. The fever, of course, is a secondary state, or consequence. But how does the inflamed part influence the whole system? It is not, Dr. C. thinks, by *pain*, since parturition and the passage of gall-stones, &c. are usually unproductive of pyrexia. It must be by irritation the result of *inflammation*, and not of spasm. Our author is often in the habit of recurring to the old subject—general fever from cerebral inflammation—for illustrations of symptomatic pyrexia. Thus if we have fever without any apparent topical affection, the latter is either masked, or the fever depends on the state of brain, as was demonstrated in a former work. Our ingenious author appears to have a horror of that old scholastic dogma—" de non apparentibus et non existentibus eadem est ratio."

" It cannot be denied, that many instances occur of a *febrile* state of system, in which, for a time at least, the existence of topical inflammation, is by no means obvious. Such cases have been called *general* or *universal* diseases, or diseases of the whole system; as if no part or organ suffered primarily or essentially more than the rest, or was immediately concerned in the production of the *febrile* state. The case of *proper* or *idiopathic fever* has been especially relied upon, in proof. A little consideration, however, will, I think, shew, that this is far from being conclusive on the subject: for, in the first place, it may be observed, that inflammation often exists where it is not suspected. It is by no means uncommon for indubitable traces of pre-existing inflammation to be met with after death, of which no suspicion was entertained during life; and that even in the most important organs, as the brain and lungs, as well as many others. This may be accounted for on different grounds; as, first, the not distinguishing sufficiently between the *primary* and essential, or *pathognomonic*, symptoms of a disease, and such as are only *secondary*, or of casual occurrence; the latter being often more striking, and more distressing to the patient, than the former; and hence both the seat and nature of the disease are liable to be overlooked. Inflammation of the liver is thus frequently not noticed, from the attention of the patient being directed exclusively to the disordered functions and feelings of the stomach, which, under the convenient name of *dyspepsia*, are considered and treated as the primary disease; often with the effect of aggravating the hepatic inflammation. In like manner, inflammation of the brain, in children more especially, is frequently ushered in by *vomiting;* and is hence considered as an affection of the stomach merely, and treated as such, perhaps by *stimulants* or *opiates*, till the occurrence of more strongly-marked cerebral symptoms denotes the true nature of the disease—a discovery that is sometimes made too late.

Another cause of inflammation being frequently overlooked, is the small share of sensibility possessed by many internal organs, which, on that account, may be actively and even fatally diseased, without being attended with pain sufficient to attract notice. The *liver* and *lungs* both furnish instances of this, but still more the *brain*. This organ, though the source of feeling to other parts, is itself among the most insensible, and its diseases in consequence the most liable to be overlooked. It is not by pain in the head, therefore, that we are enabled to judge of the degree or nature of disease in the brain, but by the disordered state of the cerebral functions. In the most acute instances of *phrenitis*, the disease often proceeds to a fatal termination, without pain in the head being at all complained of. So also, in many cases of *idiopathic* fever, the pain in the head is inconsiderable, and quite disproportionate to the other symptoms of the disease; or, if the pain in the head be at first severe, it ceases to be complained of in the more advanced stages of the disease. This may be partly ascribed to the disturbed state of the mental function in those cases; but is referrible also, in part, to the natural insensibility of the organ itself.

The case of *idiopathic* fever, therefore, that has been so much relied upon, as shewing that *pyrexia*, or a febrile state of system, may take place without the presence of inflammation, does not appear to furnish conclusive evidence on the point; because, as I have just stated, inflammation may be going on in the brain, although little or no pain be complained of at the time. It is certain, however, from the disturbed state of the *sensorial* functions (admitted to be present in every case of proper or *idiopathic* fever), that active disease of some kind is going on in the brain; and that such affection consists in inflammation, is a matter of fair inference, from the general character of the symptoms, as compared with those induced by other acknowledged inflammations in this part; while this conclusion receives all the support from dissection that could reasonably be looked for, as I have elsewhere endeavoured to shew.*" 54.

It is a thousand pities that Dr. Clutterbuck had not gone to the BAR. A better special pleader could hardly be found than he is, even in physic. But had he studied the other profession, he would have "puzzled a Philadelphia lawyer"—and that is saying a good deal!

The 5th Section is on the nature of pyrexia. The ratio symptomatum must be pretty nearly the same whether we consider the fever idiopathic or symptomatic. In running over the various phenomena presented by the vascular system, Dr. C. mentions a rather staggering fact.

"If the arteries be examined after death, in cases where the febrile action has been violent during life, their lining membrane, as well as that of the heart and larger veins, is found to be highly reddened, so as to resemble the *tunica conjunctiva* of the eye in an inflamed state, and the substance of the vessel altogether is observed to be thickened." 63.

We very much doubt whether such appearances will be found in a majority

* "Inquiry into the Seat and Nature of Fever."

of even the most violent inflammatory fevers—but at all events, such post mortem phenomena will not be found in one case out of twenty of the ordinary fevers, whether idiopathic or symptomatic, of this country. They are, therefore, very poor props for any theory. Our author, however, has too much sagacity not to perceive that "the disturbance in the condition of the brain is hardly less apparent than that observable in the organs of the circulation." No doubt of it. The nervous affection "appears to constitute, if not the first, at least one of the most important links in the chain of morbid phenomena that characterize *pyrexia*, or the febrile state." This is true as far as idiopathic fever is concerned; but we appeal to all practical men whether, in the symptomatic fevers, as in pneumonia, for example, the vascular system is not more prominently and primarily affected than the nervous? How often do we see the most intense and dangerous and local inflammations going on, with very little affection of the sensorial functions? Not so, in idiopathic pyrexia.

Dr. C. is of opinion that without the aid of SYMPATHY, or that mysterious consent between part and part, and between parts and the whole, we cannot account for the general fever that supervenes on local inflammation.

"From these and other facts, it would seem that the brain is the great organ of *sympathy*, or medium of communication, between different parts of the body, as it evidently is between *body* and *mind*." 71.

The practical drift, then, of the whole theory of our author is this:—that the presence of fever indicates some local inflammation, the seat of which we are to find out, if we can—and if so, to use local as well as general depletion. But even if we cannot detect the locality of the phlogosis, we must still treat the fever as the offspring of inflammation—namely, by depletion.

In a subsequent Section Dr. C. notices the consequences of pyrexia, namely, debility, emaciation, occasional hæmorrhage, dropsy, and tendency to inflammation in other organs besides those originally affected. These phenomena are well understood, and need not detain us here.

*Treatment of Pyrexia in General.*—After what has been said, we can hardly be surprized that Dr. C. is an advocate for the antiphlogistic and even depletory system of treatment, where there is apparent or inferred topical inflammation. He acknowledges, indeed, that—

"Instances occur, almost daily, of slight inflammations, occasioned by *cold*, (as catarrh, sore-throat, rheumatism, &c.) being successfully treated by *stimulants* of an active kind, under the denomination of *diaphoretics* or *sudorifics*; such as *camphor, ammonia*, the *hot bath*, and various others. These, by exciting the sanguiferous system, rather tend, at first, to produce or aggravate the febrile state; but if they be so managed as to induce sweating, the febrile symptoms often subside, and, at the same time, the primary inflammation which caused them. This mode of practice, however, is rather equivocal in its effects, and requires caution; for where it does not succeed in removing the primary inflammation, it is sometimes found to aggravate it. Whenever, therefore, the inflammation is seated in organs of importance to life, and which it is desirable to suppress quickly, it is hardly advisable to proceed in this way. The *sudorific* practice, however, is rendered more safe in such cases, and, at the same time, more effectual, by previous abstraction of blood, in proportion to the habit of the patient, and other circumstances." 85.

In the foregoing extract the adroit and ingenious special pleading of our author is sufficiently obvious. Dr. C. admits that, "in ordinary *continued fevers*, of a mild description, this mode of treatment (*diaphoretic*) has been, and, indeed, still is, in very general use, in preference to the more active, though unquestionably more effective means of cure by *blood-letting*." We quite agree with Dr. C. that venesection or equivalent depletion is more calculated to save than waste strength, where actual inflammatory action is going forward in the system. But the difficulty in adopting our author's methodus medendi lies in the amalgamation which he makes of idiopathic and symptomatic fevers. Bleed, purge, and sweat, say we, when inflammation is *proved* to exist;—but be cautious, or at least very sparing, of the lancet, where the proof of topical phlogosis is wanting, and where we have only Dr. C's theory to rely upon, instead of ocular or tangible demonstration.

The following passage, we think requires some comment. Speaking of topical inflammation, as for example of the lungs, he says:—

"In violent inflammations in general, wherever seated, and especially if the patient be of a vigorous or inflammatory habit, and the disease recent; attended, too, as it commonly is under such circumstances, by high *febrile* action, it is essential to endeavour to subdue this by the most active means; not only for the purpose of guarding against the consequences formerly mentioned, but also as the best means of influencing the primary disease itself: for in the most important inflammations, the part primarily affected is out of the reach of direct or topical remedies. And even where it is accessible, experience shews that local treatment is often less efficacious than the use of general remedies. Accordingly, it is found that the reduction of the *febrile action* by *blood-letting* and other *antiphlogistic* means, proves the first, and often indeed the only, step requisite to the cure; the primary disease giving way immediately that this object is attained. It is, however, important to repeat here, that this is not universally the case; for the *febrile symptoms* may wholly disappear, while the primary inflammation still continues, though, perhaps, in a mitigated form. Our care, therefore, is not to be limited merely to the removal of the *febrile symptoms* in inflammations of important organs, such as the brain or lungs, but should be continued till the *local* signs, as well as the general disorder of system, have wholly disappeared. The neglect of this, as a rule of practice, often leads to fatal mischief at a remote period afterwards; as in *pneumonia* more especially, where, if the slightest degree of inflammation be allowed to remain in the lungs, it is likely to produce disorganization, the ultimate result of which often is, *pulmonary consumption.*" 92.

Now with every respect for Dr. Clutterbuck, as an old, talented, and experienced physician, we cannot help entering our caveat against the foregoing practice. We dissent from the rule of pursuing *general* bleeding in all cases of pulmonic inflammation, or even in a majority of them, till the *local* symptoms have "wholly disappeared." Were we, for example, in delicate people, predisposed to phthisis, to carry *general bleeding* to the extent of totally annihilating the cough that attends, and, for some time, continues after, pulmonic inflammation, we would do much mischief. We would break down the tone of the constitution, and give fresh impetus to the development of tubercles—thus accelerating that fatal event, phthisis, which it ought to be our great object to prevent. No. When the sympathetic fever is reduced by general depletion, the best practice, in our opinion at least, is to remove the cough and local pains by local depletion and counter-irritation, diet, and antiphlogistic medicines. In this way we husband the powers of the constitution while we eradicate the local phlogosis and its sequels.

Dr. C. extends his principle of treatment (namely the reduction of "*febrile* symptoms" by general means) to hectic fever "attending ulceration of the lungs." We confess that, little as we expect from the boasted specifics for consumption, we should anticipate still less from this general depletory system in excavations of the lungs. We shall, however, in fairness to the author, allow himself to explain his practice.

"The case of *hectic fever*, as occurring in *pulmonary consumption*, serves to illustrate what has now been said. In the confirmed state of this disease, a cure is rarely to be expected from this or any other means. Yet much may be done to mitigate the violence of febrile action (the *hectic*); by doing which, much suffering is spared to the patient, and life often prolonged to a considerable extent. These important objects may be accomplished in many instances by bloodletting, repeated from time to time, but always in small quantities, as from three or four to five or six ounces at a time; the rule being in such cases, so to draw blood, as not sensibly to disturb the functions or feelings of the patient. When used with these precautions, and while a tolerable share of general strength still remains, the good effects of small and repeated bleedings are often strikingly displayed, by their relieving all the most distressing feelings of the patient, inducing quiet sleep, and restoring appetite if lost. And I may further add, that if the disease admits of a cure, as undoubtedly is sometimes though rarely the case, it is most likely to be accomplished by such means; and this I have seen in different instances. I consider it, however, to be essential to the success of this mode of treating *pulmonary consumption*, whether undertaken with a palliative or curative intention, that the patient should be allowed to take food, either animal or vegetable, as his habits and inclinations may lead him. And it is no small recommendation of this practice, that not only is appetite usually excited by such small bleedings, but animal food may then be taken without producing that feverish excitement that it is otherwise apt to do." 99.

Not being able to speak experimentally to this practice of drawing blood from the arm, and supplying it by means of animal food, we shall leave it to the discretion and judgment of our readers. Dr. C. when the greater part of his work was printed off, fell in with the "Elements of Medicine," by Drs. Bright and Addison. He congratulates himself on the similarity—almost identity of doctrine, as respects fever, adopted by all three. For our own parts we cannot see this identity or even close similarity; and we think our readers will be of the same opinion when they peruse the following conclusions to which Drs. Bright and Addison have come.

1st. "In every case of fever," the authors observe, "the causes producing it inflict a morbid impression upon the *nervous system*, by which the functions of that system, intellectual and bodily, are deranged, that derangement differing in degree in different cases: and in consequence of this derangement, every organ, and every function of the body, appears to be more or less disordered."

2dly. "In every idiopathic fever, this morbid condition of the *nervous system* is associated with, or presently succeeded by, a deranged or excited state of the circulation; this derangement of the circulation differing in degree in different cases, and displaying a greater or less tendency to congestion, or even inflammation, of particular parts:" and,

3dly. "In the progress of every *idiopathic* fever, the secretions or excretions of the body become deficient, vitiated, or even irritating to the parts with which they come in contact."* 115.

We appeal to our readers whether this is not the doctrine we have held for more than twenty years past. So far from fever (idiopathic) being the *consequence* of local inflammation, these gentlemen maintain that the general fever or excitement "displays a greater or less *tendency* to congestion, or even inflammation of *particular parts*"—or in other words, that the topical inflammations are the consequences, not the *causes* of the fever. This *Hibernian* coincidence between Dr. Clutterbuck and the authors alluded to, has given Dr. C. sincere gratification, and we should be very sorry to disturb the *harmony* that exists between the parties.

Having now then touched on the chief points of interest in the work, and also on the debateable topics scattered through it, we take leave of our author with the greatest respects for his talents, his zeal, and his unwearied industry in the prosecution of medical science in general, and this important path of investigation in particular.

---

* Elements of Medicine.

### Philosophy of Living. By Mr. Mayo and Dr. Tickner.

We have now two works on the "Philosophy of Living," and there appears a fair opening for a treatise on the "Philosophy of Dying." None of us are certain of life—all of us are sure to die. The last act of the drama must necessarily be tragical—and it is most important of all! We therefore hold out this tempting opportunity for some of our sage philosophers to immortalize themselves by a treatise, not like Sherlock's, "on DEATH;" but on dying—with the greatest degree of philosophy, propriety, and comfort. There is no danger of any snarling critic coming back upon the author with the posing dicta of *experience* in his mouth, to contravene the dogmas which the author may have laid down respecting the last dropping of the curtain. With whatever degree of patience the author may be listened to by the living, he is safe from all reflections by those who have put his precepts to the test of practice! But we must return to the Philosophy of Living. In our last number we took occasion to controvert some of Mr. Mayo's positions in respect to the state of the brain in sleep, epilepsy, and apoplexy. At present we are more in the commendatory mood, and mean to notice some good observations which Mr. M. has made on curvatures of the spine. He sets out, however, with a rather startling assertion.

"There is but *one disease* to which female children are liable, and that is Education; as soon as the age arrives at which they are to be artificially trained to feminineness of mind and manners and accomplishment, their strength and health are endangered. While boys are encouraged to pursue sports of increasing exertion, their sisters, whose bodily strength not keeping pace with theirs, nevertheless *requires exercise equal in proportion* for its maintenance, are forbidden all that they need."

Although the female youth is liable to the diseases of male youth, yet certainly education brings with it a host of maladies—or at least disorders. Amongst others, weakness of the spine.

"The first feature in the inquiry, which presents itself, is the almost uniform elevation and fulness of the *right shoulder and right side of the chest*, which accompanies curvature of the spine. Why this feature is not universal will be subsequently explained. But for the present let us attend to the fact of its remarkable frequency.

The principle, to which this will be traced, is thus expressed by Donald Walker,—'*The one-sidedness with which almost all the acts of life are performed, is the general cause of the greatest and most universal deformity*, and its prevention requires an equal and similar use of the other side.' Hitherto, however, the connexion between the general fact, and the common feature of spinal curvature has not been shown.

M. Coulson, in a recent work upon this subject, animadverts upon the views propounded by the late Mr. Shaw.

'In consequence,' says Mr. Shaw, 'of the alteration in the state of the shoulders being the first symptom of deformity observed, it is generally but erroneously supposed that the dorsal part of the spine is the first distorted. Indeed those who have lately written on this subject have fallen into this error, and have described the curve at the loins as the last which is formed.

In cases of diseased vertebræ there may be a curve only between the shoulders, but it invariably happens in the common lateral curvature, that where one shoulder is protruded, there is also a curve at the loins; and I have shown by diagrams in the preceding volume that this curve is not only the first formed, but that those in the upper part of the spine are consequent upon it.'

Mr. Coulson observes upon these passages, that those who describe the curvature in the loins as the last which is formed, 'are right, though they did not clearly see the cause,' and adds, that Mr. Shaw's practical observations tended, 'in spite of his hypothesis, to prove that the first curve is formed at the right shoulder.'

It is evident, therefore, that while Mr. Shaw attributed the initial curve to *the loins*, Mr. Coulson is disposed to look for it *in the back and shoulder*. The latter writer, however, puts forward, with a full general conception of its importance, the principle already quoted,—the general influence of the right-sidedness of our habits upon curvature of the back.

But neither Mr. Shaw's nor Mr. Coulson's ideas are strictly just. *The two curvatures are not successive, but necessarily simultaneous*; neither has priority; in health and strength both of these curvatures, producing serpentine flexure of the spine, continually occur, and disappear with the next change of posture. In the weak, they become permanent, and are progressively aggravated.

To trace the mischief in its progress;—

The steps by which the spine ordinarily gives way are these. The child kept at its music-stool, or books, or drawing, has a weakened and aching back. The muscles of the spine have not been invigorated by the sportive exertions, and the various changes of attitude, which nature dictates. Wearied by its task, the next change is to stand listlessly beside its governess, or in a drawing-room. What is the posture which it assumes? It is of course that which gives greatest ease to the languid muscles. The child stands with its weight supported upon one leg, the body swayed to that side, the knee of the other side bent, and the hip lowered. The limb which it uses on this occasion for support is almost always the *right* limb; for this simple reason, that it is the strongest. And the child assumes the position at all times, because it is one of change from its former more rigid position, and because, in addition, the fascial structure of the limb takes off, in that posture, some of the strain from the muscles." 127.

We shall pass over many physiological arguments in order to make room for a short extract on the remedial agents.

" 1. In the first place, a child should be broken of the habit of standing on one leg in preference to the other. It should be made to stand on both alternately. Mr. Jenkins, whose ingenious instructions have been of so much use to the youth of the last five-and-twenty years, observed to me that there was one sure receipt for producing crookedness: 'For this purpose,' he remarked, 'a child should bolt its food, and habitually stand on one leg:'—the evils proceeding from the mischievous combination of bad digestion with faulty habits of posture, are well conveyed in this apophthegm.

2. All other postures are to be avoided which tend to give predominance or inclination to one side.

3. Exercises which promote the strength of the back should be systematically employed; exercises, however, in which the limbs are not weighted, but which consist in the assumption of a succession of attitudes. Much natural grace, and ease of posture and gesture, are collaterally obtained by such practices, when judiciously selected. To mention one that is highly useful;—suppose the child to stand with its feet together, and with its face turned against the low end of a sofa the level of which reaches some way above its knees; let it then raise its hands to meet above its head, and bring them down to the horizontal line of its face; and then let it bend the body forward till the hands touch the sofa, and rise again, and repeat the exercise several times in succession.

4. The dress of a girl should not bind her chest, but should be, in fact, as light and incompressive as that of a boy, and as much indulgence in play and sportive amusement allowed, as may be consistent with the habits it is right to encourage.

5. In sitting, when already tired, the child should rest well back on her chair, the spine resting against the back of the chair, thoroughly supported by it, and the seat of the chair reaching to the bend of the knees. Her feet should be equally supported.

Such are the precautions necessary to be observed against spinal curvature; and they are sufficient to prevent it. To remove it entirely, when it exists to any great extent, is impossible; to remedy it in part during growth always practicable; to obliterate it at its commencement, not less so." 132.

---

MEDICAL SCIENCE AND ETHICS: AN INTRODUCTORY LECTURE DELIVERED AT THE BRISTOL MEDICAL SCHOOL. By *W. O. Porter*, M. D.

THIS is an eloquent address, and somewhat out of the usual routine, as it inculcates, with more than common zeal, the "HIGH TONE of MORAL FEELING," which has been presumptuously claimed by one class (a very small one) in the profession, and unmercifully ridiculed by others. There is also a large vein of *religious* feeling pervading this address—and which, indeed, seems to be gaining ground, of late, in medical writings, as compared with those which issued from the press 20, 30, or 40 years ago. Since the days of Darwin, medical men have been *suspected* of a tendency to scepticism, though, in our humble opinion, the suspicion was unfounded, and the direct charges grafted on it most false. The charge was made by two classes of society—embracing no small range—Fanatics and Hypocrites. The former class were, no doubt, sincere in their denunciations, though narrow-minded and tyrannical in their attempts to thrust their creeds down the throats of their neighbours. But the Hypocrites were far more detestable; for while the greater number of them were themselves scoffers at religion, they raised the hue and cry against their neighbours for no other reason, and from no other motives than self-interest; and a wish to raise their own reputation for sanctity on the depreciation of others. We sincerely hope that the tone of moral and religious feeling is rising, as Dr. Porter believes, in the ranks of the medical profession; but the augmenting pressure of redundancy in those ranks is not calculated to increase but to obstruct such wished for tendencies. However, as the scale of general as well as professional education is annually rising in kind as well as in amount, we anticipate much advantage from this important circumstance. Dr. Porter justly observes, that—"At this day no small proficiency is required to gain admission fairly into the profession: there is at least as much required of the general practitioner at Apothecaries' Hall at this time, as might have qualified the graduate in medicine, thirty years ago. This is honourable to the profession—it must exclude the idle and the stupid. Such will not venture into the arena."

But when, by industry and application the aspirant has passed the ordeal of Halls, Universities and Colleges, and goes forth into life with his well-earned certificates and diplomas, "he will find that he has giants in zeal and knowledge to contend with for public favour, not only in the Metropolis, but even in villages, and almost in every spot where he can plant his foot." This is a comfortable reflection, but it is one into which most people will fall, when they emerge from the schools and mix with the tide of practice and competition. The fact is, that many are daily quitting the ranks of the medical profession, from sheer inability to succeed, however hard they may have studied, however diligently they may have looked out for employment afterwards.

As we have often before remarked, there is no *cure* for the evil at all—and the only plan of *mitigating* it, is to enlarge the curriculum of education, general and professional, and also the time during which the curriculum is working. It is worse than useless to make the *amount* of knowledge very high, and leave the period of acquirement short or optional. If it be short, it is impossible to acquire the necessary knowledge *well*. The soil will be forced, or *crammed* with bad manure that will unfit it for future crops. If the period be left *optional*, it will be still worse; for then the system of *cramming* will become all but universal, and health will be ruined by the tremendous efforts to acquire the *necessary* knowledge in the shortest possible space of time. No theory was ever more erroneous, or more injurious to society and the profession, than that which would make the *examination* the whole test of elementary education. In the languages, in mathematics, and in some of the exact sciences, this tentamen might suffice; but in medicine the mode and the time of acquiring knowledge are often more important than the mere amount that may be compressed by artificial engines into the human memory.

There are a great number of excellent hints and judicious observations in Dr. Porter's address, and we recommend it to the especial notice of the Student in Medicine.

---

### TREATMENT OF CHOLERA.

ALTHOUGH the reappearance of some cases of malignant cholera in Limehouse and on board the Dreadnought has been trumpetted forth by the alarmists, the expected choleraphobia has not resulted. Sporadic cases have occurred every year since 1832, (and we believe long before that period), but the *epidemic* character of the disease has gradually subsided, and we are still of opinion that it will no more take root and become indigenous than any other *epidemic*. The influenza has never left us since 1833, and is, even now (Nov. 1837), more rife than the cholera ever was. As to contagion, the influenza exhibits the very same kind of evidence, on that point, as cholera did and does. It begins with some one member of a family, and some or all of that family become consecutively affected. This was the main argument of the contagionists, and why they did not apply it to the influenza, we cannot tell. Again, the epidemic influenzas almost invariably *travel* from east to west, the same as cholera. The influenza occasionally takes a lateral, diagonal, or even retrograde direction. So does cholera, witness its spread in Italy, after leaving Europe, and crossing the Atlantic to America. The cry is still—prepare for Cholera—take precautions to prevent its spreading! What precautions have ever prevented the diffusion of this or any other epidemic? None! All the precautions and barriers that have been employed, have invariably increased the evil by diffusing alarm and terror, the most powerful predisposing causes of cholera. We verily believe that had there never been instituted a Cholera Board of Health or a Cholera Hospital; and had a certain number of medical men been appointed to attend the poor in their own houses, and *there* supply them with food, medicines, and nutriment, there would have been infinitely less mortality in this and other countries, than under the systems that were put in force.

The more we saw of cholera, the more we were convinced that it was a serous hæmorrage from the bowels, which, after a certain or uncertain time, left the remaining blood unfitted for free circulation and the support of life—and then came the collapse, the spasms, the deadly coldness, the cessation of the pulse at the wrists, and death itself. We have heard of cholera proving fatal, without any discharge upwards or downwards. We never saw such a case,—we do not believe that such a case ever occurred. The diarrhœa serosa or cholerica, is the disease in its first or curable stage. When the blood is left black and thick by the draining away of its serum, there is small hope of recovery.

Now, whoever has had much experience in the treatment of bowel-complaints, whether in hot or in cold climates, must be well aware of the great importance of quietude, the horizontal posture, and the warmth of the bed. Attention to these points alone, with proper diet, would cure nine-tenths of the common bowel-complaints of this country, and even of the premonitory diarrhœa of cholera. If this be a correct representation, and we maintain that it is, how injurious must have been that system which dragged the unfortunate poor from their homes to cholera hospitals, under the influence of terror, exposed to cold—and what was as bad or worse, to the jolting and motions of a hackney-coach, which inevitably increased the diarrhœa, and, no doubt, precipitated the state of collapse. All this was done under the false theory that the epidemic was a dire contagion, worse than the plague of the East! Should the epidemic revisit this Metropolis, we would propose a simple, and we believe an efficient plan for checking its ravages amongst the POOR—for the rich will take care of themselves. It is this: let each parish engage one, two, or three medical men to visit the habitations of the poor, so as to detect the premonitory diarrhœa, and prescribe for the patients at their own houses—the prescriptions to be procured at the nearest chemists in order to save time, the signature of the medical attendant being a voucher for payment by the parish. The same voucher might also serve for any article of wholesome nourishment procured from the nearest source of supply. The whole expense, on this plan, would be very trifling; and we are perfectly satisfied that it would prove more efficient than all the sums lavished on Cholera Hospitals, fumigations, and the other parts of the machinery put in motion upon epidemic visitations. It may be objected that there will still be a certain portion of the poor so completely destitute as to require removal from their hovels to some asylum. It was a disgrace to England, and to the Governors of Hospitals, in this country, to see their gates shut against cholera patients, while the same institutions were thrown open on the Continent. The unmanly fear of *contagion* brought this, among numerous other evils in its train! And after all, this ignoble precaution completely failed in its object, for the epidemic leaped over the petty and panic-stricken barriers of our hospitals, and laughed to scorn the foolish foundation on which they were built. It is to be hoped that a better regime will be adopted should the enemy ever again knock at our gates.

In respect to the treatment, we may again observe that the premonitory diarrhœa is best checked by confinement to bed, external warmth, perfect quietude, and *starvation*. We have seldom found much difficulty in stopping the diarrhœa, when the case was recent, and the blood not too far deprived of its serous constituents—and that by opiates, cordials, and the common astringents. Dr. Graves, of Dublin, has, however, published a paper on the internal use of acetate of lead and opium, which, in his experience produced the most beneficial effects. We certainly have used this astringent, amongst others, but not with the marked superiority which attended its administration in the hands of Dr. Graves. We give, however, his formula:—

"During the preceding months of May and June, I had treated several cases of diarrhœa in fever with large doses of the acetate of lead, according to Dr. Bardsley's plan, and I had had frequent opportunities of admiring the efficacy of this salt in checking profuse alvine discharges. Just as Mr. Ryan died, and while my mind was filled with regret at our failure in his case, I was called by Dr. Percival Hunt to see a lady in Nassau-street, labouring under dysentery; I advised the free use of acetate of lead, and with marked success. Immediately after, I saw a case of cholera still in the stage of premonitory diarrhœa, or rather just passing from the bowel complaint into the fully formed disease. I tried the acetate of lead boldly, and with the happiest success. Thus encouraged, I applied this new method of treatment in every case to which I was called, and I was employed both night and day in visiting cholera patients, and every hour gave me additional proofs of the efficacy of the remedy. My formula was as follows:—

℞ Acetatis Plumbi, ℈j.; Opii., gr. j, M. fiat secundum artem massa in pilul. xii. dividenda.

The premonitory diarrhœa has almost invariably stopped by taking one of these pills, at first every hour, and as the stools became less frequent, every third or sixth hour, according to circumstances. When the vomiting, spasms, and the state of collapse had begun, it was necessary to give a pill every quarter of an hour: after a couple of hours the effect of the pills became perceptible, in a diminution of the serous evacuations upwards and downwards; then the pills were given only every hour, and as the symptoms yielded they were given less and less frequently, and could in general be laid aside altogether before twenty-four hours. In some it was found necessary to give the acetate of lead in solution, combined with a little vinegar and minute doses of acetate of morphia. Minute doses of opium were useful; anything of large doses hurtful. Mr. Parr, the able and respected apothecary of the Meath Hospital, was saved by acetate of lead, after the usual astringents, combined with large doses of opium, had been fully tried. He was found by me to be sensibly under the narcotic influence of opium, but the peculiar symptoms of cholera had not been thereby checked. Many took more than forty grains of the acetate of lead in twenty-four hours; it usually darkened, or even blackened, the alvine discharges, before they ceased altogether. Were I to enumerate all the cases of violent cholera that yielded to this treatment, I would be led into a tedious but not an uninstructive detail."—*Medical Gazette.*

Since the publication of Dr. Graves's paper, Dr. Venables has stated that the salts of lead were tried on a large scale by him, at the suggestion of the late Sir D. Barry, and failed. We have no doubt that all remedies will fail if not early employed, and with the auxiliaries which we have pointed out. If the auxiliaries are promptly employed, in conjunction with *remedies*, these last will be found amongst those in ordinary use.

P. S.—Since the above was written, we have received a paper from Mr. John Paterson, of Aberdeen, respecting the epidemic cholera, as it occurred there and at Colleston, a fishing-village near that place. We are unable to spare space for Mr. P.'s paper, but may state that he derived great benefit from venesection in the early stages of the cholera. He describes the destructive effects of the panic occasioned by the doctrine of contagion. When he went, by order of the BOARD OF HEALTH, to Colleston, he found all work suspended—"the villagers being prevented by the *public authorities*, from entering the neighbouring towns with their fish for sale!" These were some of the blessed effects of the contagion doctrine, and of BOARDS OF HEALTH. Mr. P. endeavoured to dissipate the fears of the people, and had well nigh succeeded, "had not his exertions been met by the troublesome, and, he firmly believes, useless enactments of the BOARD OF HEALTH." "*Useless*" indeed! They were every where highly *injurious*. The only good they ever did, was in the character of scavengers—and for one life saved by carrying away filth, ten were sacrificed by the terror of contagion, and the misery and want produced by the cessation of intercourse and occupation! May we never live to see another Cholera Board.

---

### CHANGES PRODUCED IN THE NERVOUS SYSTEM BY CIVILIZATION, &c., &c. By *Robert Verity*, M. D.

THE marrow of this little work, which is rather an introduction than a finished essay, may be contained in a very short compass. The principle which Dr. Verity elaborates, has long been acknowledged. It is this—that whatever structure or function, in the human body, is called into greater activity than other structures or functions, there will be a corresponding acquisition of power in the former. Thus the arm of the gold-beater is seen to be stronger, in proportion, than his leg—and the same is evident in the coachman, because these people exercise the upper much more than the lower extremities. The *eye* of the watchmaker sees and detects minute objects much more keenly than that of the musician, whose *ear* is far more sensitive to sounds than that of the watchmaker. The tongue of the cook or gastronome, has a better sense of taste than that of the sailor or soldier. The same principle is seen in the intellectual functions. The poet who has toiled for years in his garret or closet, would ill support the tremendous burthens of the coal-heaver, and the *latter* would experience no small difficulty in constructing an epic poem. This principle might be extended to almost any length—and from the body it may be transferred to the mind—from the physique to the morale.

Our author appears to anticipate a regularly increasing *perfectibility* in the human race, by the steady advance of intellectual activity. We much doubt whether this anticipation is supported by the "philosophy of history," or even by the "evidence of physiology." It is very true that the *Press,* and the diffusion of knowledge may, and probably will, prevent the *dark ages* from ever returning; but there are many sources of national decay which literature, science, and intellectual activity may be unable to control. Dr. Verity must know that excess in intellectual exertions, like inordinate corporeal labour, has a strong tendency to wear out the organ of the mind, and the mind itself. There requires no great research for numerous examples of this. Intellectual excitement may be pushed beyond the salutary point in nations as well as individuals, and premature decay may be the consequenence. The following is all very fine upon paper.

"It has never been found in history, that it was difficult for the animal powers to become less and more mitigated in their expression, but the difficulty has been for the higher powers to become developed equal to the sustainment of continuous civilization. It is not enough to let the former be effaced by inaction, to die of an atrophy, but their activity must be replaced by positive development of intelligence, by something pregnant with prospective advancement.—But the last northern migrations, which took place in the first centuries of the Christian era, from their magnitude and duration, and the form and complexion they have impressed on our European world, demand a more marked attention, as well as a more detailed analysis of the physiological development of the kind of society into which they settled down. Such seems the advance and position of human affairs at present, that, perhaps, it may emphatically be said, these northern migrations are, indeed, to be the last. It is difficult to contemplate an historical spectacle of more grandeur and importance than the movement of so many races and nations adventurously precipitating themselves upon the entire face of the old civilized world—careering to and fro all around the circuit of the Mediterranean shores, as if fetched out of unknown regions, and guided by an Almighty hand, to accomplish a salutary, settled purpose in the destinies of mankind. For upon this great event the fortunes of the human race have turned. It was the collision and intermixture, one with the other, of the many and various elemental powers of human society, qualities of blood and of character, languages and institutions, which subsequently gave birth to new and more vigorous forms of social life. For, from this transfusion of so many native and untainted elements into the languishing and dying current of Roman civilization, cemented together and controlled by the commingling influence of the Christian principles, was formed the general European stock, which has divided itself into the different nations of modern times, having, however, from the similar circumstances of their origin, many characteristics in common. It was an amalgamation of that astute, selfish, and indomitable will of the old Roman—that practised and well balanced understanding in political matters, with the rich intellectual freshness and more generous and loftier moral energy peculiar to the Teutonic and Scandinavian races; and, in a physiological point of view, we must never forget that these distinctive classes of qualities, with many others arising from greatly different organic temperaments, were equally reflected in the tissues and structures of the physical body. Where the admixture partook more fairly of the different elements, the fusion has taken place more rapidly and with more thorough effect; the fermentation and final adsettlement (so to speak) was sooner completed, and there has been evolved from the inward and amalgamating action, a more perfect and compact unity of national character; and it will be found that the political and domestic institutions of a nation, as it approached this type, will have become the sooner mature, vigorous, and progressive."

Nations may, and no doubt will, advance far beyond the present point of intellectual activity and capacity; but we much doubt whether the acmé of mental improvement will prove a complete check to various causes of degeneration and decay. Look at France in the revolution, when she presented a scene that threatened national extermination! Look at Spain now—three centuries more barbarous than in the days of Cervantes!

As for the physiological progression towards perfection, we apprehend that the argument founded on the increase of longevity in modern times, is fallacious. Our organization is not a bit *more* perfect now than it was two centuries ago—we think it is not so robust. Our diseases have not decreased in number, but they have in intensity; and we have learned to treat them more skilfully, and prevent them more effectually. Yet still the increase of longevity is to be chiefly traced to the withdrawal of numerous causes of disease—to more cleanliness and comfort—to more temperate habits—to cultivation and draining of the soil, and to a thousand other points of Hygiene, which were imperfect amongst our forefathers. In former times, fevers, small-pox, agues, and various acute diseases carried off thousands, where they now only destroy hundreds; whereas, in modern times, we have hosts of nervous and dyspeptic affections that do not perhaps materially shorten the natural range of human existence, while they render life very miserable. But we need not pursue the subject farther, as Dr. Verity has promised a work in which the principles here slightly developed will be applied to medical science directly. If Dr. Verity would not be offended by our freedom, we would advise him to involve or clothe his ideas in a much smaller number of words. It is by no means easy to make out Dr. Verity's meaning upon all occasions; and no fault is more dangerous, in an unpractised author, than a diffuse style, which, like too great a dilution of wine with water, destroys at once the flavour and the relish.

---

A TREATISE ON THE INFLUENZA OF 1837, AS OBSERVED AT BIRMINGHAM. By *Peyton Blakiston*, M.A., Med. Lic. Cantab. 8vo. pp. 60. Birmingham, October, 1837.

THE author attended about 200 cases, chiefly belonging to a public institution, and has here given an analytical or clinical report of 100. The labour of observing and recording them must have been very considerable, perhaps more than was necessary, for less than half, the number would have illustrated the epidemic quite as well as the centenary of cases. Dr. B. has not "travelled from his record" by adverting to the peculiarities of preceding or accompanying seasons, nor by instituting comparisons between the epidemic of 1817 and those of other years. These tasks he leaves to others. The author throws these cases into three sections; first, containing cases as types of the disease in persons previously healthy; secondly, cases of influenza modified by pre-existing disease; third, a general analysis of symptoms and treatment. It is only from the third section that we shall extract any materials for this short article.

1. *Age.*—Although no age was exempt from the epidemic, yet the largest proportion of cases occurred between 20 and 50 years of age—that is, in the prime of life. This coincides with the experience of Louis and Andral in respect of fevers generally.

2. *Diet* seemed to have nothing to do with the predisposing causes.

3. *Locality.*—By far the greater number of patients had been resident in Birmingham for years. The houses of the patients were, generally, wholesome and well ventilated. The site of the town is elevated, and its surface very irregular, so that it is well drained. It is, however, cold, and bronchial irritation is prevalent. Our author could trace no connexion between the locality and the disease.

4. *Habits.*—Thirty cases occurred among the wealthier classes, and 70 among the humbler, so that people of all intermediate ranks were included. The author was not able to discover any modification of the disease from variety of habit, manners, or avocations.

4. *Contagion.*—On this point our author has not been able to make up his mind, and therefore "has no opinion to offer on the subject."

State of the different Organs.

As no dissections were obtained, Dr. B. cannot, of course, offer any pathological phenomena, excepting such as may be inferred from symptomatology. There was no fatal case, except where the influenza was complicated with some other disease.

*Pharynx.*—In a large majority of cases, there was redness of the fauces and tonsils. *Tongue.* In some, even of those who died, the tongue presented no abnormal appearance. In 45 cases, however, it was covered with white, in 39 with a soft yellow fur. *Nausea or vomiting* occurred in almost every case. In many, bile was ejected. *Loss of appetite* was almost universal. In 60 cases out of 100 there was no *thirst.* In the others, it existed more or less. In 70 cases the bowels were confined. In 26 cases the bowels were natural. In four they were disordered. The *urine,* in a great many cases, was thick, as in common catarrh. In respect to the action and sounds of the *heart,* none were abnormal unless they had been so previously. The *pulse,* in a great majority of cases, was small, feeble, and sharp at the onset—soon becoming soft, and continuing feeble for a long time. In the majority of cases it did not exceed 100 in the minute. When the pulse was above 80, the skin was dry and more or less hot. In some weakly patients there was a constant sensation of chilliness. In 40 cases there was no increase of temperature. *Cough* existed in all cases, shewing itself from the first till the seventh day. In all cases it was violent for at least two days; in many cases it was prolonged for some weeks, assuming a convulsive character. *Expectoration.* When this became copious and easy, the pain and uneasiness of the cough were much diminished. When the patients were in health previously to the attack, the expectoration was clear mucus; when the mucous râle existed, it was very viscous. In those who were the subjects of chronic catarrh, the expectoration was muco-purulent. Most patients felt a dull pain under the sternum upon coughing, and many complained of constriction across the chest. The chest sounded clear on percussion, in every case where no pneumonia or asthma was combined with the influenza. Respiration was also clear and vesicular. In 75 cases, no râle was audible; in three cases, slightly sibilous and sonorous; in 18 cases, these râles were well-marked. There were symptoms of derangement of the nervous system in all cases, varying from a slight headache, lassitude giddiness, &c., to excessive headache, great prostration of strength, and occasional delirium.

*Treatment.*—In simple cases the treatment was very inert. The symptoms were watched, and the functional derangements of each organ ameliorated by mild means. When congestion or inflammatory action of the mucous membrane of the lungs was rendered obvious, blisters, sinapisms, or even leeches, were employed; as also the ætherial tincture of lobelia inflata combined with ipecacuan wine, and occasionally with soda.

The following are the conclusions to which our author comes from a careful statistical analysis of 100 cases, besides a general observation of many others.

"1. The influenza, as observed at Birmingham, is an affection of the nervous system, with its concomitant derangements in the organs of digestion, circulation, &c., commonly known under the name of *nervous fever;* accompanied, *throughout its whole course,* by irritation of the pulmonary mucous membrane.

2. This irritation, not unfrequently, amounted to congestion, and even to inflammation.

3. The influenza was modified by pre-existing disease, more particularly by chronic bronchitis, the subjects of which were rendered liable to the acute form of that disease.

4. Neither locality, previous habits, or diet, acted as pre-disposing causes.

5. In simple, uncomplicated cases, mild treatment alone was sufficient.

6. When bronchitis was present, counter-irritation, and large doses of ætherial tincture of lobelia, repeated at short intervals, seemed useful.

7. Venesection was always counter-indicated.

8. It was often necessary to have recourse to diffusible stimulants, at the commencement of the complaint, and to administer tonic medicines in an early stage of it.

9. It only proved fatal in those cases where the persons it attacked had been enfeebled by old age or chronic disease."

As there is scarcely a disease or disorder the causes of which do not make their first impression on the *nervous* system, so Dr. Blakiston may be justified in terming the epidemic influenza a *nervous* disease. Indeed it must be acknowledged that the epidemic visitations of this malady have always been remarkably characterized by depression and derangement of the nervous system, as contradistinguished from purely inflammatory affections of the respiratory apparatus. We think that our author is entitled to the thanks of his brethren for this accurate and minute statistical analysis of the various phenomena exhibited in the different organs and functions during the course of influenza.

---

### THE FLORA OF JAMAICA, OR A DESCRIPTION OF THE PLANTS OF THAT ISLAND. By *James Macfadyen*, M.D.

THIS is really a most praiseworthy production, and reflects very high credit on the talents and industry of Dr. Macfadyen. The present volume contains a minute botanical description of the plants of Jamaica, arranged in the various natural orders, from the Ranunculaceæ to the Leguminosæ; and to each description are appended valuable remarks on the general properties, uses, and natural history of the plants. We are promised the second volume in the course of the present year, and a series of illustrations of such plants as are new or may not have been previously figured.

When completed, the work will be a most useful compendium to the medical practitioner who visits Jamaica, or indeed any other tropical climate, and to such we very strongly recommend it. For the purpose of facilitating the pursuit of the less practised botanist in discovering any particular plant, there will be an appendix in the second volume, containing an enumeration of the genera according to the Linnæan or artificial system, and also an essay on the geographical distribution of the species. Dr. Macfadyen tells us that the work has occupied a great portion of his leisure during the residence of twelve years in the island.

"I have carefully," says he, "examined the characters of every plant within my reach, and compared my own descriptions with those of preceding botanists. I have visited a considerable portion of the island, so that I have had opportunities of studying the peculiarities of the flora of each district. The nature also of my occupation, as a medical practitioner, has been of some advantage, as, in my almost daily rides, I have had an opportunity of watching each plant during its period of flowering and perfecting its fruit."

As a sample of the contents of the work, we select the description of the plant which yields a cocoa or chocolate nut.

"THEOBROMA.—Calyx of 5 sepals; petals 5, fornicate; nectary urceolate, with 5 horns; filaments 5, each with 2 anthers; style filiform; stigma 5-parted; capsule 5-celled, without valves; seeds in a buttery pulp; albumen 0; cotyledons thick, oily, corrugated.

THEOBROMA CACAO. *Chocolate Nut.*—Leaves entire, smooth, ovate, oblong, acuminate.

*Hab.*—Cultivated.

*Fl.*—Throughout the year.

[Then are enumerated the various synonymes.]

This is a tree of moderate height (12 to 16 feet); the trunk upright; the bark brownish; and the wood light and porous. Leaves rather large, lanceolate. Flowers small, reddish, inodorous, numerous, scattered over the trunk and branches. The fruit is a coriaceous capsule, partaking somewhat of the form of a cucumber, reddish, and marked with 10 grooves externally; internally there are 5 compartments, filled with a gelatinous acid pulp, enveloping the seeds. When ripe, the external surface is either a deep red or a yellow, and the seeds rattle in the capsule. The tree bears leaves, flowers, and fruit throughout the year. The usual seasons, however, for gathering the fruit are the months of June and December. The seeds are 20 to 30 in number; when fresh, they are of a reddish brown colour. They quickly lose their power of vegetation, if taken out of the capsule; but, if kept in it, they preserve it for a considerable time."

Appended is a good account of the natural and commercial history of the tree and of its seeds; of the mode of preparing chocolate; and of some interesting particulars respecting the use of this substance as an article of diet.

---

REMARKS ON THE TREATMENT OF FRACTURES OF THE LOWER EXTREMITIES WITHOUT THE AID OF SPLINTS. With Cases. By *John F. Burke*, Member of the Royal College of Surgeons. 8vo. stitched, pp. 39. London, 1837.

MR. BURKE is a strong admirer of Mr. Radley, which we are not. He dedicates this pamphlet to him, and offers it as a proof that, indifferent as the profession has been to the discoveries of the great "anti-splinter," he, at all events, appreciates them.

"Although," says Mr. Burke enthusiastically to Mr. Radley, "upwards of two years have elapsed without producing any visible proof that other surgeons have adopted your views, yet let us hope that your endeavours to do away with the old system of tormenting fractures, have been silently and surely working their proper effect in more quarters than one; and that the period is not far distant when your zeal for the advancement of Surgery shall meet with a better tribute than is here offered you by—Your very obedient Servant,

JOHN F. BURKE."

When he descends from the warmth of dedication to the sober task of developing his views, Mr. Burke again deplores the Cimmerian gloom which would seem to have settled on Mr. Radley's doctrines.

"It is not a little surprising that, since the publication of Mr. Radley's remarks in 'The Lancet,' respecting his plan of treating fractures without the application of splints, the subject has been allowed to fall to the ground without further notice by members of the medical profession, except in the meetings of some medical debating societies, where, indeed, the theory has been occasionally discussed, but generally without the aid of practical information, which alone can test its merits." 5.

The surprise will be proportioned to the faith of the reader. If he has become a convert to the opinions and the practice of the hater of splints, of course he will be astonished that others have not grown converts too. But, if made of incredulous stuff, and impervious to the new lights, he will feel no sort of surprise at all. This is just our case. Our readers need not be told that we are not proselytes of Mr. Radley's. The evidence he gave was not enough to satisfy us. We predicted at the time that the fate of all discoverers awaited that gentleman. He was doomed, we felt sure, to experience neglect from his generation. Even Galileo and Harvey were laughed at. How much more then must Mr. Radley be so. Two years have passed. A disciple of Mr. Radley's testifies to the indifference displayed towards him. Alas! the course of merit, like that of true love—"never does run smooth." Mr. Radley is too much a-head of his century. He has worked for posterity; we hope it will pay him.

We do not intend to rip up the case "Radley *versus* Splints." For it, and for the arguments for plaintiff and defendant, we refer to former numbers of this Journal, as well as to other periodicals. We shall now content ourselves with chronicling the views and exposing the doctrines of Mr. Burke.

"It must be allowed," says this Gentleman, "that the plan of curing fractures without the aid of splints is by no means new, inasmuch as we are daily in the habit of seeing fractures of the lower extremities laid in junks, without any splints whatever being previously put on them; and perhaps it may be argued that no better application than junks, contained within some soft substance, can be adopted for the purpose of retaining fractures of the lower extremities in their proper positions. Supposing, then, that this be granted, what do we concede, but that splints (properly so called) are unnecessary for keeping fractured bones in apposition? It is the very point to which Mr. Radley has attempted to draw the attention of surgeons; and having once done away with the idea of the *necessity* of using splints in the treatment of fractures, we shall have to consider whether their employment, so far from being requisite, be not, in many instances, absolutely hurtful, by exciting the action of the muscles of the limb to which they are applied, and by always proving, to a certain extent, galling to the feelings of the patient, and particularly so during the first week after the occurrence of fracture." 7.

Mr. Burke misapprehends the question, and consequently does not rightly put the argument. The point at issue is not whether the mere instrument called *splints* should be employed in the treatment of fractures, but whether *forcible extension* which splints are a common, but only one, mode of applying, should be had recourse to. Mr. Radley says forcible extension is injurious and unnecessary—ninety-nine out of a hundred surgeons reply that, when properly understood and used, it is neither injurious nor unnecessary. To the latter it is no argument to contend that junks are admitted to be useful. When a fractured leg is put up in junks, the limb is first forcibly extended until it is brought as nearly as possible to its natural form and length; then the junk is secured, and by *lateral compression* it retains the limb in the state to which it has been brought. This is a very different affair from laying a broken limb upon a pillow, and expecting that the muscles will behave themselves.

The cases related by Mr. Burke are two—one of fractured thigh *in a child*—the other of both bones of the leg, a little above the ankle. It must be admitted that such cases are not very conclusive. Fractures of the thigh in children are well known not to require much extension, and fractures of the leg a little above the ankle scarcely call for any. We may conceive that *such* cases, with moderate attention, may do pretty well on a pillow. Mr. Burke, indeed, observes, that:—

" I had subsequently several other cases of fracture of the lower extremities (which it is unnecessary to relate), all of which I treated in the same manner, and all of which did remarkably well, *without the slightest shortening or deformity of the limb.*"

But he gives no particulars on which we may ground an opinion.

Mr. Burke has been singularly fortunate or unfortunate in his observation of the results of the treatment by splints.

" We have only," says he, " to take notice of the numerous cases of fracture that are frequently to be met with, in which, after the ordinary treatment by splints, the ends of the fractured bones will be found riding upon each other, and encircled by an enormous callus, which must, in a great measure, impede the proper action of the muscles which pass over or are at all connected with it. It may possibly be asserted, that this is not in consequence of the application of splints, but of the restlessness of the patient; but, if this plea be again admitted, we have yet to consider how far this restlessness is induced by the pressure of splints, even supposing them to be productive of no other bad effect." 8.

Now the object of applying a splint or splints (and, if properly applied, they will fulfil that object) is to maintain apposition of the portions of bone, and rest. How this is to produce an enormous mass of callus we leave to physiologists—how it is to occasion restlessness on the patient's part, we leave to casuists to determine. We give up both points. Nay, with some portion of experience, with a fair share of observation, we confess that, setting aside the explanation, we have not had the luck of noticing the facts. We have neither seen restlessness, riding of bones, nor masses of callus, result from splints at all. But, by a curious infelicity, we have seen, or we fancy we have seen, all from the want of splints, or, what is practically almost the same thing, from their mal-application by ignorant people. But we will not argue the question further. We are unwilling to laugh—the mass of our readers might resent our being serious. We will content ourselves with simply stating the details of Mr. Burke's method, and then leave the anti-splint plan to its fate. We may say to it, as one of the Lake Poets said to one of the Lake Poems—

Go, little book, from this my solitude!
I cast thee on the waters; get thee forth!
And if, as I believe, thy tone be good,
The world will by-and-bye detect thy worth.

But to bring Mr. Burke's plan of proceeding under the notice of our readers.

" It now only remains for me to say a few words respecting the mode of placing a fractured limb on pillows, in order to preserve it in as quiet a state as possible. If the surgeon in attendance have a predilection for bandages, of course the many-tailed bandage must first be laid on the pillow; and, if previously wetted with the saturnine lotion, or any other that may be preferred, will be found to lie more neatly than if used in a dry state; besides which advantage, it will not afterwards shrink on the application of a lotion, and occasion more pressure than is requisite. The bandage having been properly applied, the pillow, which should be placed on a mattress, is to be drawn round the limb by tapes in the usual way, and these tapes subsequently fastened to either side of the bed, in order to obviate the chance of lateral motion. The pillow is then to be tacked firmly to the mattress in several points, indeed wherever there appears any possibility of its being moved; and if the feathers be firmly pressed together on either side of the limb before the tapes are tightened, a degree of support is afforded, which has all the advantages, without the galling unpleasantness, of splints. Indeed, those who have not tried this plan will, I am confident, be surprized at the resistance made by the pillow, even to a sound limb; and, be it remembered, a fractured one is not capable of much effort. A folded sheet or table-cloth may be laid neatly under the pillow at the bend of the knee, for the purpose of relaxing the muscles, in the same manner as when the inclined plane is used; and as the pillow should not meet over the leg, another folded cloth, saturated with some cooling lotion, should be loosely laid between its sides. Care should be taken that the pillow used be broad enough to rise sufficiently high above the limb to prevent any pressure from tapes or bandages, and also that it be long enough to project a little beyond the foot. A piece or two of tape, sewn on to each side of the pillow at its extremity, thus enables the surgeon to draw it round the foot instead of the foot-board commonly in use, and should the foot incline a little too much to one or the other side, a compress placed between it and the pillow immediately rectifies its position.

Where the femur is fractured, two pillows will be found necessary; and these, in order to render their surface perfectly smooth, should be united by a folded cloth of sufficient thickness, laid over them. Should a child be the subject of fracture, a good-sized bolster will be sufficient for all purposes."

---

ELEMENTS OF CHEMISTRY, INCLUDING THE RECENT DISCOVERIES AND DOCTRINES OF THE SCIENCE. By the late *Edward Turner*, M.D. Sixth Edition, enlarged and revised. By *Justus Liebig*, Professor of Chemistry in the University of Giessen; and *Wilton G. Turner*. 8vo., pp. 410. Taylor and Walton.

THE death of Dr. Turner has created a void in the great medical schools of London, which, even in such schools, is not easily supplied. Amiable as a man, eloquent as a teacher, and eminent as a chemist, he enjoyed not only extended fame, but his image was enshrined in the hearts of his pupils. Long may it live there, though its prototype is taken from them.

The Elements of Chemistry of Dr. Turner have been prized as a succinct and valuable *exposé* of the state of the science at the period of their publication. We say at the period of their publication, for chemistry is a science peculiarly progressive, and in the interval between two editions of a popular work, such advances may have been made as to render the later edition very different in many respects from its predecessor.

It is hardly necessary for us in the present instance to do more than quote the advertisement of the editors to the edition before us—the sixth—of the present work. That advertisement will explain the plan adopted by the publishers, and the alterations made and contemplated by the editors.

"The conduct of the work has been entrusted to Professor Liebig and Mr. Wilton G. Turner. The department of Inorganic Chemistry has been prepared by Mr. Turner. The description of the Imponderable Agents was left nearly ready for the press by the Author, and the only additions necessary were the insertion of a few recent discoveries. In the remainder of this section, the Editor has endeavoured to effect the improvements which he was aware had been contemplated by his brother. He has, consequently, treated of the Elements and their Compounds, under the three heads of History, Preparation, and Properties. An arrangement which, it is hoped, will not only increase the utility of the work as a book of reference, but will add to the clearness of its descriptions. The old chapter on Crystallography was formed on the views of Haüy; it has been re-written, to suit the present state of the science.

The department of Organic Chemistry requires to be entirely re-written, owing to the many new facts which have been discovered since the publication of the last edition. This Professor Liebig has kindly undertaken. In the volume about to be presented to the public will therefore be developed, for the first time, the views of this distinguished chemist on that department of the science to which he has devoted his particular attention. A departure from the mode of publication originally announced has consequently been occasioned. The work will now consist of Three Parts. Part II., containing the Metals and Salts, will be ready by the first week in November, and with Part I. will embrace that portion of the subject required for Lectures previous to April. Part III., consisting of Organic Chemistry, will be published early in 1838. In consequence of the arrangement adopted by Professor Liebig, it has become necessary to transfer to his section the description of several substances formerly treated under Inorganic Chemistry.

In a Preface by the Editors, which will accompany the Third Part, will be given a more particular account of the changes adopted in this edition. An Appendix will be added, containing any discoveries that may have been made during the publication of the work."

We would only say, on parting, that we do not approve of this method of publishing works in parts. The public have smarted pretty severely from this ad captandum device, and we are much mistaken if publishers do not shortly find that it is grown musty, and will not take.

Of the work itself we can speak in high terms. It is the best manual of chemistry for a student which exists in our own tongue. We are in this only re-echoing the public decision. We recommend the present edition in the strongest terms to our readers.

---

The British Medical Almanack, 1838; with a Supplement. Edited by *William Farr*. To be continued annually. 12mo., pp. 224. London, 1838. Price Three Shillings.

We are glad to perceive that the "Almanack" has weathered the storm that wrecked the "Annals of Medicine." Mr. Farr is the editor of both, or, rather, is the editor of one and *was* the editor of the other.

We have only space at present to say a very few words. The present Almanack appears to us to have both the faults and the same merits as its predecessor. Its faults are—a dogmatic tone—a rather *queer* assortment of matter—confident opinions on subjects of medical policy which are in themselves debateable and debated. Its merits are—good arrangement—a large mass of useful information—and an exact spirit, highly conducive to the advance of knowledge. It would be unfair not to add that the merits preponderate vastly, and that the work is one which deserves encouragement. We recommend it to our readers.

